# Relationship between behavioural coping strategies and acceptance in patients with fibromyalgia syndrome: Elucidating targets of interventions

**DOI:** 10.1186/1471-2474-12-143

**Published:** 2011-06-29

**Authors:** Baltasar Rodero, Benigno Casanueva, Juan V Luciano, Margalida Gili, Antoni Serrano-Blanco, Javier García-Campayo

**Affiliations:** 1 Department of Psychology. Centro Rodero, Clínica de Neurociencias, Santander, Spain; 2 Rheumatology Clinic. Clínica de Especialidades, Santander, Spain; 3 Department of Psychiatry. Parc Sanitari Sant Joan de Déu, & Fundación Sant Joan de Déu, Sant Boi de Llobregat, Barcelona, Spain; 4 Department of Psychiatry. Institut Universitari d'Investigació en Ciències de la Salut (IUNICS), University of Balearic Islands, Spain; 5 Department of Psychiatry. Miguel Servet University Hospital. University of Zaragoza. Instituto Aragonés de Ciencias de la Salud (IACS), Spain; 6 Red de Investigacion en Actividades Preventivas y Promocion de la Salud RD 06/0018/0017, Research Network on Preventative Activities and Health Promotion

## Abstract

**Background:**

Previous research has found that acceptance of pain is more successful than cognitive coping variables for predicting adjustment to pain. This research has a limitation because measures of cognitive coping rely on observations and reports of thoughts or attempts to change thoughts rather than on overt behaviours. The purpose of the present study, therefore, is to compare the influence of acceptance measures and the influence of different behavioural coping strategies on the adjustment to chronic pain.

**Methods:**

A sample of 167 individuals diagnosed with fibromyalgia syndrome completed the Chronic Pain Coping Inventory (CPCI) and the Chronic Pain Acceptance Questionnaire (CPAQ).

**Results:**

Correlational analyses indicated that the acceptance variables were more related to distress and functioning than were behavioural coping variables. The average magnitudes of the coefficients for activity engagement and pain willingness (both subscales of pain acceptance) across the measures of distress and functioning were *r = *0.42 and 0.25, respectively, meanwhile the average magnitude of the correlation between coping and functioning was *r = *0.17. Regression analyses examined the independent, relative contributions of coping and acceptance to adjustment indicators and demonstrated that acceptance accounted for more variance than did coping variables. The variance contributed by acceptance scores ranged from 4.0 to 40%. The variance contributed by the coping variables ranged from 0 to 9%.

**Conclusions:**

This study extends the findings of previous work in enhancing the adoption of acceptance-based interventions for maintaining accurate functioning in fibromyalgia patients.

## Background

Fibromyalgia (FM) syndrome is a chronic rheumatologic disorder of unknown aetiology that affects between 2 and 4% of the general population [1]. Some environmental familial factors, such as learned strategies for coping with problems in life, have been pointed to as intrinsic parts of the pathogenesis of fibromyalgia [2]. The traditional approach to treatment typically focuses on symptom reduction through medical management or self-management approaches, often within the context of multidisciplinary pain management programs [3].

Behavioural and cognitive-behavioural treatments, which are included in these programs, are based on the idea that modifying an individual's responses to his or her condition will reduce disability and suffering from chronic pain. Researchers had paid attention to the fact that although chronic pain could lead to dysfunction among some individuals, others seem to adjust relatively well to the ongoing experience of pain; additionally, these researchers tried to identify the factors that promote adaptive functioning in the face of pain. Along these lines, a great deal of research has examined the range and efficacy of patients' "coping" strategies [4]. It has been assumed that an individual's choice of coping strategies will determine his or her adjustment to chronic pain, and research has focused largely on identifying healthy strategies. Unfortunately, research using coping strategies has more readily identified detrimental--rather than specific and adaptive--coping responses. For example, coping responses such as guarding or resting have often shown a strong positive association with disability and distress [5,6].

Therefore, researchers and clinicians have begun to embrace emerging psychological theories that discuss acceptance in relation to the effects of aversive thoughts, moods, or sensations. Acceptance-based interventions attempt to teach clients to feel emotion and bodily sensations more fully and without avoidance and to notice the presence of thoughts without following, resisting, believing, or disbelieving them [7]. However, it is understood that experiential avoidance is a process in which an individual attempts to change the form or frequency of a private event that he or she is unwilling to experience. Although experiential avoidance might be effective in the short term, in the long term, it seriously limits quality of life. Most of the actions of patients with chronic pain are aimed at avoiding painful sensations and emotions as well as thoughts or memories associated with pain, but paradoxically, as has been widely documented [8,9], avoidance behaviour leads to disability.

A great deal of research supports the role of pain acceptance in the daily functioning of people with chronic pain. In clinical samples, the acceptance of pain is associated with less pain, distress and disability [10-12] and with greater psychological wellbeing [13]. In treatment outcome studies, acceptance-based methods are associated with improved emotional, psychosocial and physical functioning and with reduced healthcare use [14-17].

The traditional medical approach uses strategies (e.g., encouraging wellness-focused strategies and discouraging illness-focused strategies) [18] to alleviate or avoid symptoms. In contrast, acceptance-based interventions, rather than attempt to eliminate unwanted experiences, help the individual to identify valued directions, start to act in those directions and, thus, to follow a meaningful life. For this purpose, patients are taught how to make willing contact with and tolerate the experience of pain or other distressing events that might appear, without attempts to control them [19]. Coping with pain is directly trying to change pain, and what the person feels and thinks about pain. Acceptance of pain is directing efforts towards functioning and living; acceptance is "coping" with life.

McCracken and Eccleston [20,21] each found that acceptance of pain accounted for much more variance in measures of patient functioning--including disability, work status, depression and pain-related anxiety--than did a measure of cognitive strategies. Both studies used the Coping Strategies Questionnaire (CSQ) [22]; however, the CSQ has been observed to be more heavily weighted towards the measurement of cognitive rather than behavioural coping strategies, and this represents a limitation [23]. Cognitive coping instruments depend on patient memory to gauge accurately what the patient usually does to cope. It is possible that patients may place more weight on their most recent coping efforts when rating their "usual" coping responses. Memory is also mood-dependent, and because pain can influence mood, it can likewise affect memory [24]. To deal with these concerns, the utilisation of measures of behavioural coping efforts that are readily observable, such as rest, medication or exercise, is highly recommended.

The primary aim of this study was to replicate and extend the findings of previous studies using the Chronic Pain Coping Inventory (CPCI) [18], which is an inventory that is focused on behavioural strategies. In addition to having been validated in a sample of Spanish patients with fibromyalgia [25], this questionnaire has explained unique and significant variance in measures of adjustment when compared to the CSQ [6,26]. It was expected that the acceptance-based measures would continue to show greater utility in comparison with the behavioural coping strategies in predicting important aspects of patient distress and functioning. Furthermore, the CPCI will allow us to observe differences between acceptance and behavioural strategies and to elucidate the targets of intervention in pain management.

## Methods

### Settings and Participants

The study sample consisted of 167 patients who were recruited from the 41 primary healthcare centres in the city of Zaragoza, Spain, during the year 2010.

To be included in the study, patients were required to fulfil several inclusion criteria: (1) be between 18 and 67 years old; (2) be able to understand and read Spanish; (3) meet the American College of Rheumatology criteria for primary FM [1]; and (4) have been diagnosed by a Spanish National Health Service rheumatologist. Exclusion criteria included the following: (1) diagnosis with a severe Axis I psychiatric disorder (dementia, schizophrenia, paranoid disorder, or abuse of alcohol and/or drugs) or a severe Axis II disorder (personality disorder) that, from the clinician's point of view, might prevent them from following the study protocol; and (2) refusal to participate. The study questionnaires and protocol were approved by the Ethical Committee of the regional health authority, and the patients signed a consent form attesting to their willingness to participate.

### Measures

#### Demographic and Pain-Related Variables

Each participant was interviewed and provided information about a number of demographic and pain-related variables including age, work status, time diagnosed with FM, medications and other medical treatments.

#### Visual Analogue Scale (VAS)

The Visual Analogue Scale (VAS) consists of a 10 cm long straight line whose extremities represent the limits of pain intensity (from none to unbearable). Patients estimated the pain intensity experienced between 0 and 10 at the time that they were interviewed.

#### Physical symptoms

The number of comorbid physical symptoms was obtained from a standardised symptom checklist [27]. This self-report checklist instructs participants to indicate whether they experienced each of the 75 symptoms for at least 3 months over the past year. A score was obtained by totalling the affirmative responses to all 75 symptoms. Sample symptoms include dry eyes, shortness of breath, dizziness, irregular heartbeat, tingling in the extremities, urinary urgency, and coughing spells.

#### Chronic pain acceptance questionnaire (CPAQ)

The Chronic Pain Acceptance Questionnaire (CPAQ) is a 20-item inventory designed to measure the acceptance of pain [12]. There are two principal factors measured by this questionnaire: activities engagement and pain willingness. All items are rated on a 0 (never true) to 6 (always true) scale. Nine items measuring pain willingness were reverse-keyed. Following the scoring procedure, a single total score was calculated based on the nine reverse-keyed items and the other eleven items measuring activities engagement. The maximum possible total score is 120, with a higher score indicating better acceptance. The Spanish version of the CPAQ showed sound psychometric properties (α = 0.79-0.86) and good test-retest reliability (intraclass correlation coefficient 0.83) [28].

#### The Chronic Pain Coping Inventory - 42 (CPCI-42)

The Chronic Pain Coping Inventory (CPCI) [18] was originally a 65-item self-report questionnaire; based on recent analyses, it has been shortened to 42 items [29]. It asks patients to rate the frequency of use of behavioural and cognitive strategies over the previous week. It has the same CPCI-65 strategies, which are grouped into the following eight subscales: Guarding, Resting, Asking for Assistance, Relaxation, Task Persistence, Exercise/Stretch, Seeking Social Support and Coping Self-Statements. This instrument was translated and validated into Spanish by our team. Reliability coefficients were adequate based on the current data (α = 0.65-0.82) and test-retest reliability (intraclass correlation coefficient 0.76) [25].

#### Hospital Anxiety and Depression Score (HADS)

The HADS [30] is a self-report scale designed to screen for the presence of depression and anxiety disorders in medically ill patients. It is appropriate for use in both community and hospital settings and contains 14 items rated on 4-point Likert-type scales. Two subscales assess depression and anxiety independently. The HADS has been validated in a Spanish sample [31] and has demonstrated good reliability and validity. The test-retest reliability presented correlation coefficients above 0.85, and the internal consistency showed satisfactory coefficients α = 0.86 (anxiety) and α = 0.86 (depression).

#### Medical Outcome Study Short Form 36 (SF-36)

The Medical Outcome Study Short Form 36 (SF-36) is a 36-item instrument designed to measure general health status and health-related quality of life [32]. One item assesses perceived change in health status, while 35 items examine eight generic domains in both physical and mental health. The eight domains include Physical Function, Physical Role, Bodily Pain, General Health, Vitality, Social Function, Emotional Role and Mental Health. Scores in each subscale range from 0 to 100 with higher scores indicating better health status. The Spanish version of SF-36 has been shown to be reliable with good construct validity (α = 0.78-0.96) [33].

#### Fibromyalgia Impact Questionnaire (FIQ)

The Fibromyalgia Impact Questionnaire (FIQ) is a 10-item self-report questionnaire developed to measure the health status of fibromyalgia patients [34]. The first item focuses on the patient's ability to perform functional activities. In the next two items, patients are asked to circle the number of days in the past week that they felt good and the number of days that they missed work. Finally, the last seven questions (ability to work, pain, fatigue, morning tiredness, stiffness, anxiety, and depression) are measured with the visual analogue scale. This instrument has a translated and validated Spanish version [35] that showed good psychometric properties (α = 0.82-0.86) and good test-retest reliability (intraclass correlation coefficient 0.74) [28].

### Statistical methods

#### Sample size

The sample size was calculated based on the population that suffers from FM in the region of Aragon, which according to previous studies [1] can be estimated in 36,000 patients out of a total of 1,200,000 inhabitants that live in Aragon. With a confidence level of 95% and an estimated error of 5% based on previous studies [20,21], a sample of 167 patients was necessary for an adequate power calculation [36]. EPIDAT 3.1 was used to calculate the sample size.

#### Analysis strategy

Prior to analyses, a factor analysis was performed to determine if there was any overlapping between scales. Results suggested excluding four SF-36 subscales: first, the Physical Role and Bodily Pain subscales and, second, the Emotional Role and Mental Health subscales because of their overlap with the FIQ and HADS scales, respectively. Next, to compare and contrast our results, we followed the same steps as McCracken and Eccleston's previous papers [20,21]. First, a correlation analysis with the Bonferroni correction (*α*= 0.05/n) was used to assess the relationship among acceptance subscales, coping scores, and measures of pain and functioning [37]. Additionally, Cohen's criteria [38] were taken into account to evaluate the substantive significance of correlations (large correlations are those > 0.50, medium correlations are from 0.30 to 0.49, and small correlations are from 0.10 to 0.29). Then, two sets of hierarchical regression analyses were performed to investigate combined and unique relations of acceptance and coping scores with measures of functioning. The criterion variables included pain, number of symptoms, FM impact, general health, vitality, anxiety, depression and physical and social functioning. In the first set of analyses, the eight coping variables were tested as predictors for entry at the predictive model, and then the two acceptance scores were tested for entry *(p <*0.05 to enter, *p *> 0.10 to remove). In the second set of analyses, the order of entry was reversed; first, the acceptance scores were tested for entry, and then the coping scores were tested. Together, this regression method is designed to show which variable set accounts for the largest increment of unique variance in the measures of pain and functioning. Condition indices were inspected to flag excessive collinearity in the data (a condition index over 30 suggests serious collinearity problems). All statistical analyses were performed using the SPSS 15 statistical package.

## Results

### Characteristics of the participants

The study sample consisted of 167 patients (90.4% women and 9.6% men), aged 19 - 67 years (50.6 years, SD = 9.9 years); all of them were self-described as from the Caucasian ethnic group. On average, the patients had been suffering from fibromyalgia for 12.3 years (range 1 - 40 years), and 19.7% had been granted an invalidity pension.

### Correlational analyses between study measures

Results from correlational analyses of acceptance subscales, coping scores, and measures of pain and functioning are shown in Table 1.

**Table 1:** Correlations of Acceptance scales and Coping strategies with Pain, Number of symptoms, Fibromyalgia impact, General health, Anxiety and Depression (*N *= 167)

	Activity engagement	Pain willingness	Pain (VAS)	Number of symptoms	Physical functioning	General health	Vitality	Social functioning	Fibromyalgia Impact	Anxiety	Depression
*Acceptance- measures*											

Activity engagement			-0.42^** c^	-0.20**	0.44^** c^	0.41^** c^	0.33^** c^	0.50^** c^	-0.62^** c^	-0.42^** c^	-0.53^** c^

Pain willingness	0.28^** c^		-0.32^** c^	-0.14	0.34^** c^	0.35^** c^	0.28^** c^	0.17*	-0.32^** c^	-0.31^** c^	-0.27^** c^

*Coping strategies*											

Guarding	-0.42^** c^	-0.29^** c^	0.28^** c^	0.16*	-0.46^** c^	-0.30^** c^	-0.34^** c^	-0.35^** c^	0.49^** c^	0.25^** b^	0.28^** c^

Resting	-0.45^** c^	-0.37^** c^	0.34**	0.22**	-0.29^** c^	-0.28^** c^	-0.39^** c^	-0.39^** c^	0.54^** c^	0.35^** c^	0.41^** c^

Asking for assistance	-0.35^** c^	-0.19*	0.23^** a^	0.15*	-0.33^** c^	-0.24^** b^	-0.33^** c^	-0.18*	0.42^** c^	0.17*	0.22**

Relaxation	-0.06	-0.25^** b^	0.01	0.30^** c^	-0.18*	-0.14	-0.07	-0.14	0.10	0.05	-0.02

Task persistence	0.49^** c^	0.17*	-0.34^** c^	-0.10	0.35^** c^	0.24^** b^	0.21**	0.30^** c^	-0.37^** c^	-0.30^** c^	-0.37^** c^

Exercise/Stretch	0.08	-0.11	-0.01	0.11	-0.00	0.06	0.04	0.00	0.05	0.00	-0.07

Seeking social support	-0.14	-0.21**	0.17*	0.09	-0.20**	-0.07	-0.10	0.01	0.20**	0.21**	0.02

Coping self-statements	0.10	-0.08	-0.10	0.08	-0.02	0.21**	0.06	0.08	-0.06	-0.11	-0.24**

*Note*: Pain was assessed with a 100 mm visual analogue scale, Number of symptoms with standardised symptom checklist, General functioning with some of the SF-36 subscales, the Fibromyalgia impact with the FIQ, and Anxiety and Depression with the HADS.

*p < 0.01; ^** ^*p *< 0.05; Bonferroni-corrected p values; a = 0.003125 (0.05/16); b = 0.0028 (0.05/18); c = 0.00069 (0.05/72).

Both acceptance scores were correlated with task persistence but negatively correlated with guarding, resting and asking for assistance. Furthermore, pain willingness was negatively correlated with relaxation and seeking social support.

The acceptance subscales were significantly correlated with almost all nine of the measures of pain and functioning in the expected direction. The average magnitudes of the coefficients for activity engagement and pain willingness across the measures of distress and functioning were *r = *0.42 and 0.25, respectively.

Forty-three out of 71 of the correlations between the coping scores and measure of pain and functioning were significant, at *p *< 0.05. The average magnitude of the significant correlation was *r = *0.17. Guarding, resting and asking for assistance were reliably associated with poorer functioning in nine out of nine measures including greater pain, number of symptoms, anxiety and depression. Seeking social support was also related to greater problems with functioning, reaching significance in four out of nine measures. In only 10 out of 71 instances did coping scores corre-late with measures of distress and functioning in a way that suggested a positive relationship. Task persistence was associated with better functioning in eight out of nine measures. Coping self-statements were associated with two out of nine measures. Contrary to our expectations, exercise/stretch did not show any correlation with the diverse variables, and relaxation was associated with a greater number of symptoms and worse physical functioning.

After the Bonferroni correction for multiple tests, fifty-one correlations still remained significant. Forty-six of these correlations fulfil the most stringent criteria used (*p*= 0.00069; 0.05/72). Within this criterion, it is noteworthy that there were sixteen out of 19 possible correlations between the acceptance scores and measure of pain and functioning, which demonstrate the importance of the acceptance measures. Only four correlations met with the second corrected p-value (*p*= 0.0028; 0.05/18), and, finally, only one correlation complied with the less stringent criteria (*p*= 0.003125; 0.05/16).

### Hierarchical regression analyses

Table 2 shows the results of the first set of regression analyses. Resting and task persistence showed significant contributions to six of the nine regression equations. Guarding made significant contributions to four of the nine regression equations, coping self-statements and relaxation contributed to two, and exercise and seeking social support contributed to one. In general, resting predicted greater pain, fibromyalgia impact, anxiety, and depression and predicted worse vitality and social functioning. Guarding predicted a greater impact on general function and worse general health and physical and social functioning. Relaxation predicted a greater number of symptoms and worse general health. However, coping self-statements predicted better general health and less depression. Exercise contributed to better general health, and seeking social support contributed to better social functioning. Furthermore, it is worth noting that task persistence made significant contributions to six of the nine regression equations, and its predictions were associated with better wellbeing, including less pain, impact, anxiety, and depression, and better physical and social functioning. Asking for assistance was the unique subscale that did not make any contribution. Both acceptance scores were selected together in four out of nine equations; otherwise, activity engagement was selected as a predictor of the number of symptoms, impact, vitality, social functioning and depression, but pain willingness alone did not predict any variable. The sums of variance increments attributed to all selected coping variables ranged from 7.4 to 37%. The variance increments for the acceptance scores ranged from 3.2 to 12%. Across the seven equations, the average variance contributed by coping and acceptance were 20 and 8%, respectively.

**Table 2:** Hierarchical regression analyses examining prediction of Pain, Number of symptoms, General functioning, Fibromyalgia impact, Anxiety and Depression after controlling for Coping strategies

Predictor	β (final)	Δ*R*^2^	*p *<	*R*^2^
*Pain*				

1. Task persistence	-0.15	0.11	0.001	

Resting	0.09	0.051	0.01	0.16

2. Activity engagement	-0.22	0.050	0.01	

Pain willingness	-0.19	0.030	0.01	0.24

*Number of symptoms*				

1. Relaxation	0.26	0.074	0.001	0.07

2. Activity engagement	-0.17	0.032	0.01	0.10

*Impact*				

1. Resting	0.25	0.28	0.001	

Guarding	0.17	0.058	0.001	

Task persistence	-0.01	0.028	0.01	0.37

2. Activity engagement	-0.42	0.11	0.001	0.49

*Physical functioning*				

1. Guarding	-0.24	0.18	0.001	

Task persistence	0.13	0.064	0.01	0.24

2. Activity engagement	0.26	0.066	0.001	

Pain willingness	0.16	0.024	0.05	0.33

*General health*				

1. Guarding	-0.18	0.083	0.001	

Coping self-statements	0.29	0.089	0.001	

Relaxation	-0.18	0.033	0.05	

Exercise/Stretch	0.13	0.025	0.05	0.23

2. Pain willingness	0.23	0.067	0.001	

Activity engagement	0.20	0.032	0.05	0.33

*Vitality*				

1. Resting	-0.30	0.16	0.001	0.16

2. Activity engagement	0.22	0.043	0.001	0.20

*Social functioning*				

1. Resting	-0.17	0.13	0.001	

Task persistence	0.05	0.041	0.01	

Guarding	-0.14	0.021	0.05	

Seeking social support	0.16	0.028	0.05	0.22

2. Activity engagement	0.35	0.084	0.001	0.30

*Anxiety*				

1. Resting	0.13	0.11	0.001	

Task persistence	-0.08	0.032	0.05	0.15

2. Pain willingness	-0.21	0.056	0.01	

Activity engagement	-0.22	0.031	0.05	0.23

*Depression*				

1. Resting	0.22	0.15	0.001	

Task persistence	-0.08	0.070	0.001	

Coping self-statements	-0.18	0.041	0.01	0.26

2. Activity engagement	-0.35	0.080	0.001	0.34

*Note*: Pain was assessed with a 100 mm visual analogue scale, Number of symptoms with standardised symptom checklist, General functioning with some of the SF-36 subscales, the Fibromyalgia impact with the FIQ, and Anxiety and Depression with the HADS.

In these analyses, the eight Coping scale scores were tested for entry (p < .05) and removal (p > 0.10) on initial steps based on statistical criteria. After Coping scores meeting criteria were selected, the Acceptance scores were similarly tested for entry.

Table 3 includes the results of the second set of regressions in which the acceptance scores were entered prior to the coping scores. Both acceptance scores were selected together as predictors in six out of nine equations. Activity engagement was selected alone as a predictor of a number of symptoms, including social functioning and depression. In each case, acceptance predicted better functioning. Resting and guarding were selected as significant predictors in three out of nine equations with both predicting poorer functioning. It is remarkable that there was not any significant coping predictor for anxiety. The variance contributed by acceptance scores ranged from 4.0 to 40%. The variance contributed by the coping variables ranged from 0 to 9%. Across the nine equations, the average variance contributed by acceptance was 22%, while the average variance contributed by coping was 4.7%.

**Table 3:** Hierarchical regression analyses examining prediction of Pain, Number of symptoms, General functioning, Fibromyalgia impact, Anxiety and Depression after controlling for Acceptance of pain

Predictor	β (final)	Δ*R*^2^	*p *<	*R*^2^
*Pain*				
1. Activity engagement	-0.25	0.17	0.001	

Pain willingness	-0.22	0.041	0.01	0.21

2. Task persistence	-0.17	0.022	0.05	0.23

*Number of symptoms*				

1. Activity engagement	-0.17	0.040	0.01	0.04

2. Relaxation	0.26	0.067	0.01	0.10

*Impact*				

1. Activity engagement	-0.42	0.38	0.001	

Pain willingness	-0.06	0.024	0.05	

2. Resting	0.23	0.071	0.001	0.40

Guarding	0.17	0.021	0.05	0.49

*Physical Functioning*				

1. Activity engagement	0.33	0.22	0.001	

Pain willingness	0.16	0.038	0.01	0.26

2. Guarding	-0.25	0.054	0.01	0.31

*General health*				

1. Activity engagement	0.23	0.17	0.001	

Pain willingness	0.26	0.060	0.01	0.24

2. Coping self-statements	0.25	0.043	0.01	

Guarding	-0.17	0.024	0.05	0.30

*Vitality*				

1. Activity engagement	0.20	0.13	0.001	

Pain willingness	0.11	0.031	0.05	0.16

2. Resting	-0.27	0.057	0.01	0.21

*Social functioning*				

1. Activity engagement	0.41	0.24	0.001	0.24

2. Resting	-0.19	0.030	0.05	0.27

*Anxiety*				

1. Activity engagement	-0.31	0.15	0.001	

Pain willingness	-0.25	0.057	0.01	0.21

*Depression*				

1. Activity engagement	-0.38	0.26	0.001	0.26

2. Resting	0.23	0.035	0.01	

Coping self-statements	-0.19	0.038	0.01	0.33

*Note*: Pain was assessed with a 100 mm visual analogue scale, Number of symptoms with a standardised symptom checklist, General functioning with some of the SF-36 subscales, the Fibromyalgia impact with the FIQ, and Anxiety and Depression with the HADS.

In these analyses, the two Acceptance of pain scores were tested for entry (p < .05) and removal (p > 0.10) criteria. The eight Coping scale scores were tested for entry or removal on subsequent steps based on the same statistical criteria.

## Discussion

The purpose of this study was to compare the acceptance of chronic pain with behavioural coping in predicting adjustment to chronic pain and, in the process, to replicate and extend McCracken and Eccleston's earlier papers [20,21]. The results of the present work can be summarised as follows: a greater acceptance of chronic pain was associated with less pain, symptoms, fibromyalgia impact, anxiety, and depression as well as with better general health, vitality and physical and social functioning. Regarding behavioural coping strategies, guarding and resting were consistently associated with a greater fibromyalgia impact and, individually, with less healthy functioning. Regression analyses revealed that in the first and more conservative model, acceptance added to the variance explained, independently of coping, all of the outcomes, with variance increments averaging 8% (compared to 20% for coping). When the model was reversed, many of the coping effects diminished, and acceptance continued to independently predict outcome on all adjustment measures with variance increments averaging 22% (compared to 4.7% for coping).

Although this study confirmed that acceptance of pain can still account for more variance than various measures of behavioural coping, in a range of important measures of distress and patient functioning, the results of this study were slightly different from those of other studies [20,21]. There are two possible reasons for these differences. First, previous studies used cognitive coping questionnaires, and it may be possible that behavioural coping predicts both distress and functioning better. Another possible reason is that fibromyalgia is a chronic disorder characterised by a large number of symptoms. Previous work has pointed out the possibility of fibromyalgia patients showing fewer acceptance scores than other pain conditions [28], so this would also explain the lack of a greater difference between measures. Indeed, the acceptance mean scores for other pain conditions were 47.8, 49.0 and 50.4 [15,17,39], which are substantially different from our fibromyalgia sample, where the mean score was 40.3.

Previous research has shown on more than one occasion that CPCI has three well-defined groups [18,29]: the illness-focused group (guarding, resting and asking for assistance), the wellness-focused group (task persistence, relaxation, exercise/stretching and coping self-statements), and a neutral group (seeking social support). Most of our results are concordant with previous studies, but there are also some incoherent results. Relaxation was associated with a greater number of symptoms and worse general health; the exercise/stretch strategy only contributed to explaining one positive variable; and the coping self-statements only contributed to explaining two. Unfortunately, these results are usual when presumably adaptive strategies are sought, and a series of studies has shown they are only weakly or inconsistently related to functioning [40-42]. Furthermore, as in previous studies regarding coping strategies [25,29], our results show types of patient behaviour that lead to more suffering and poor functioning and not the types of patient behaviour that lead to less suffering and better functioning. For example, strategies such as guarding or resting seem to be reliable in predicting poor wellbeing. However, there is one behavioural coping subscale that predicted good functioning consistently--task persistence--and this is also in agreement with previous studies [18,29].

From a traditional medical approach, it is assumed that good strategies need to be identified and targeted in order to improve outcome treatment. Although well intended, such approach shows that it is difficult to conclude which type of strategies are adaptive without taking into account the context. It might be appropriate to interpret the strategy in light of the intention, avoidance or non-avoidance. Strategies aimed at reducing symptoms (e.g., relaxation) or fibromyalgia impact (e.g., as resting or guarding) as well as at avoiding unwanted private thoughts, feelings and sensations are generally associated with a poorer general functioning. Conversely, strategies that are focused on proceeding despite symptoms--tolerance for symptoms--paradoxically are associated with less symptoms, less fibromyalgia impact, less distress, and better general functioning. Therefore, it seems that in chronic conditions, where the psychological area is of great importance, the acceptance-based approach is highly recommendable.

The results obtained here are limited mainly due to the cross-sectional design of the study: correlation methods cannot unambiguously infer a causal relationship. Additional research should compare acceptance scores and coping methods in an experimental pain situation [43]. Second, the list of coping questionnaires validated in Spanish is limited. The domain of coping was sampled with the contents of only one inventory, the CPCI. Other inventories exist that conceptualise pain coping strategies in different ways with potentially different results. Furthermore, to obtain a more representative sample, specifically of the male gender, it would be desirable for subsequent studies to use larger samples. Finally, another possible limitation could be that the sample in this study was a non-treatment-seeking population whose pain duration was longstanding. It is therefore possible that this sample of fibromyalgia patients may have responded differently from others.

## Conclusions

The main conclusion of the present study is that the coping-behaviours strategies often targeted within treatments have not been shown to be related to outcomes as predicted. Additionally, acceptance measures may offer more utility in guiding treatment. It seems wise, therefore, for targets of intervention to focus not only on what the clients must accomplish but also on how one can encourage them to accomplish the necessary tasks. Acceptance-based interventions seem to promote a motivational context that makes it easier for the client to move forward. There are ongoing studies in this area trying to re-appraise some of the coping responses defined as adaptive within current psychological frameworks [44-46]. Additional research is needed to clarify the processes underlying the acceptance-based strategies.

## Abbreviations

FM: Fibromyalgia; CSQ: Coping Strategies Questionnaire; CPCI: Chronic Pain Coping Inventory; VAS: Visual Analogue Scale; CPAQ: Chronic Pain Acceptance Questionnaire; HADS: Hospital Anxiety and Depression Score; SF-36: Medical Outcome Study Short Form 36; FIQ: Fibromyalgia Impact Questionnaire.

## Competing interests

The authors declare that they have no competing interests.

## Authors' contributions

BR, MG, JGC, ASB and JVL conceived the study design. BR and JGC collected the data, BC and BR conducted the statistical analysis, and all authors interpreted the results, drafted the manuscript, and read and approved the final manuscript.

## Pre-publication history

The pre-publication history for this paper can be accessed here:

http://www.biomedcentral.com/1471-2474/12/143/prepub
